# 

**Published:** 1892-02-20

**Authors:** 


**Notices of Births, Deaths, and Marriages**, are inserted in this column at a charge of 2s. 6d. for an announcement not exceeding four lines.

**DEATH.**

At Eastbourne, Nurse ELISE FIELD, aged 38, from diphtheria, contracted while nursing a child whose life she is believed to have been the means of saving. (844)

### NOTICE TO CORRESPONDENTS.

All MS., Letters, Books for Review, and other matters intended for the Editor should be addressed THE EDITOR, THE LODGE, PORCHESTER SQUARE, LONDON, W.

The Editor cannot undertake to return rejected M.S., even when accompanied by stamped directed envelope.

**Notice to Advertisers and Subscribers.**

All Advertisements, Orders for Copies of THE HOSPITAL, and Business Communications should be addressed to The Publisher, *not to the Editor*, THE HOSPITAL, 140, Strand, W.C.

The Cost of Subscription, post free, is as follows:—Per week, 1½d.; per quarter, 1s. 8d.; per half-year, 3s. 3d.; per annum, 6s. 6d. Foreign:—Per annum, 8s. 8d.; payable, in all cases, in advance.

**"Burdett's Hospital Annual," 1891—1892.**

Third year of publication. 600 pp. Boards, gilt lettering. A most complete guide to British and Colonial Hospitals, Dispensaries, Nursing and Convalescent Institutions, and Asylums. Price 3s. 6d. Published by THE HOSPITAL (Limited), 140, Strand, W.C.

**NOTICE.—The Index for Vol. X. (ending September, 1891), is on sale at the Office of this Journal, price 2½d., post free.**

### GENERAL CHARITIES.

A bazaar has been arranged for May 11th and 12th, in aid of the **BOYS HOME** in Regent's Park Road, which is at present much in need of funds for re-asphalting the play-ground. It is to be called "The United Service Bazaar," and the stallholders will be clad in military and naval uniforms, a novel and becoming toilet. The Boys' Home being a charity which should appeal to all classes, it is to be hoped that many will take the opportunity now offered to come forward and materially help it by their presence at the bazaar, or by sending contributions of money or work to the Secretary, the Boys' Home, Regent's Park Road, N.W.

### SCRAPS AND GLEANINGS.

THE Council of the Hospital Sunday Fund have received a further donation of £100 from Mr. Ludwig Mond, F.R.S.

THE late Miss Lavinia Warrington has left £7,000 for the enlargement and alterations of the Torbay Hospital and Dispensary.

IT is proposed to hold a bazaar in the autumn to raise money for the building fund and general expenses of the Ulster Hospital for Women and Children.

A NEW wing has been added to Stroud Hospital containing, amongst other necessary additions, rooms for the isolation of infectious cases, a paying ward, and new committee-rooms.

THE Royal Ear Hospital, Soho, which has just completed its seventy-fifth anniversary, is about to be enlarged and otherwise adapted to meet the increased requirements of patients.

IT was decided at the annual meeting of the Royal Bath Hospital and Rawson Convalescent Home, at Harrogate, to make a special appeal to the towns in the West Riding for funds to reduce the building debt of £11,397.

LORD CARRINGTON, as Chairman of the festival dinner to be held at the Hotel Metropole on February 24th, has issued an appeal for funds on behalf of the East London Hospital for Children and Dispensary for Women.

THE Bishop of Southwark has raised a fund of £529, which it is proposed to devote to the purpose of securing admission into convalescent institutions for those of the South London poor who are in urgent need of such assistance.

AN old Crown servant has just died at Spital, near Windsor—Mrs. Mary Ann Wellbelove, in her ninety-second year. In her younger days she was in the service of George IV., at Cumberland Lodge, and she had seen two Royal Jubilees.

BURNLEY is to have a new Union Infirmary. It is to be built on the pavilion principle. Two pavilions will be erected first, one for men and one for women, with a central administrative block, together with sundry wards for special cases.

THE Harrogate Cottage Hospital presents a satisfactory report. The largest number of patients since its opening (1,121) were treated during the past year. The financial condition of the institution is sound and the charity in good working order.

ON Sunday morning, February 7th, the Rev. H. W. Webb-Peploe, Vicar of St. Paul's, Onslow Square, in his sermon appealed for funds for the Brompton Consumption Hospital. The collection, including some annual subscriptions, amounted to £60.

THE annual dinner of the French Hospital and Dispensary will take place at the Hotel Métropole, on the 20th inst., under the presidency of M. Waddington, French Ambassador, who will be supported by the Lord Mayor and the Sheriffs of London.

THE miner, Johann Latus, of Mystowitz, is said to have awoke from his four months' sleep. He can recall no sensation experienced during that time, and is astonished to find how long he has been unconscious. He refuses all nourishment except milk.

BY command of Her Royal Highness the Princess of Wales, several copies of Canon Fleming's sermon, "Recognition in Eternity," have been forwarded to the British Home for Incurables at Clapham for the inmates by the publishers, Messrs. Skeffington and Son.

THE Children's Hospital, Bradford, has received the sum of £132 as the result of the charity ball given in December last. The report for 1891, being the first complete year of work in the new Hospital, is most satisfactory as to the number of patients treated, but the Hospital is in need of funds to pay off the building debt.

THE accounts of St. George's Hospital for the quarter ending the 31st of December, 1891, are satisfactory, although £2,000 of Consols had been sold. This sale of stock has been applied towards the expenses in connection with the additions and alterations now in progress, and to the purchase of a lease for £675.

THE ladies of Blackpool have decided that a grand bazaar shall be held in 1893 in aid of the funds of the new infirmary. At a meeting at the Town Hall, the Lady Mayoress made an eloquent appeal, and the proceedings throughout were most pleasant and enthusiastic. £58 has been collected by ladies from penny subscriptions alone.

A NEW wing has been added to the North Ormesby Cottage Hospital, Middlesborough, containing a large room for out-patients, with a dispensary, consulting-room, and dressing-room. On the first floor are nurses' day and bed-rooms, a bath-room, and other conveniencies. Mr. J. M. Bottomley was the architect, and has expended £,006 on the work.

THE thirty-first annual report of the Children's Hospital, Birmingham, shows that the number of in-patients last year was 841, out-patients 11,999, casualties 832, and home patients 22. The detention rate was 21·3 days, the death-rate 10·4 per cent., the cost of maintenance of patients 2s. 9d. per week, and of officers 8s. 3d. per week. The income was £80 less than the expenditure.

A UNIQUE and profitable entertainment in aid of the Rosehill Hospital (Babbicombe) for Sick and Incurable Children took place at the Bath Saloon, Torquay, on February 3rd. The tableaux, five in number, were pictures of a flower carnival, set in a garland of roses. Redhead's cantata, "The Flower Pilgrims," was given during the interludes. Then came the battle of flowers, followed by the well-known farce, "Ici on parle Francais."

IN the year 1782, when influenza appears to have been universal, an unknown bard sent the following lines to the *European Magazine* for June of that year:—

"Influenza haste away!
Cease thy baneful empire here!
Boast no longer of thy sway,
Cease dominion o'er the year!
Radiant Sun, exert thy pow'r,
On the wings of Zephyr come,
Dart thy beams and rule the hour,
Health and Beauty then shall bloom!"

### THE STUDENT OF MEDICINE.

[All communications for this Supplement should be addressed to THE EDITOR of THE HOSPITAL, at 140, Strand, with the words, "Students' Column," written in the left hand top corner of the envelope.]

### APPOINTMENTS.

C. Brooks, M.R.C.S., L.R.C.P., appointed House Surgeon to the Liverpool Northern Hospital, vice R. D. Mothersole, resigned. W. C. Bull, M.B. Camb., F.R.C.S., appointed Aural Surgeon to St. George's Hospital. R. C. M. Colvin-Smith, B.A.Camb., M.R.C.S., L.R.C.P., appointed House Physician to the Victoria Hospital, for Sick Children, vice A. Gale, M.R.C.S., L.R.C.P. G. Dean, M.A., M.B., C.M.Aberd., appointed Assistant to the Pathologist and Curator of the Museum, Aberdeen Royal Infirmary. A. F. Dimmock, M.D.Durh., M.R.C.S., re-appointed Medical Officer of the Harrogate Cottage Hospital. E. H. Gonin, M.B.Edin., appointed House Physician to June 30th, 1892, to the City of London Hospital for Diseases of the Chest, Victoria Park, vice Dr. E. C. Williams, resigned. L. J. Hobson, M.D.Lond., B.S., F.R.C.S.Eng., appointed Honorary Consulting Physician to the Royal Bath Hospital and Rawson Convalescent Home, Harrogate. J. F. Jordan, M.B., M.R.C.S., appointed Assistant Surgeon to the General Hospital, Birmingham, vice W. F. Haslam, F.R.C.S. J. Lea, B.A., M.B., B.C., appointed Assistant House Surgeon to the Liverpool Northern Hospital, vice S. J. Palmer. A. W. Loveridge, M.R.C.S.Eng., L.S.A., appointed Honorary Surgeon to the Newport and County Infirmary, vice Dr. Marsh, resigned. F. L. Norris, M.B., C.M.Glas., appointed Junior House Surgeon to the Borough Hospital, Birkenhead. F. N. Ozanne, L.R.C.P.Lond., M.R.C.S., re-appointed Medical Officer to the Harrogate Cottage Hospital. S. J. Palmer, M.B., M.R.C.S., appointed House-Physician to the Liverpool Northern Hospital, vice C. Brooks. J. Panting, M.A.Cantab., M.R.C.S., L.R.C.P., appointed House Surgeon at the Middlesex Hospital. H. M. Richards, M.B., B.S.Lond., appointed House Physician to University College Hospital. N. E. Roberts, M.B., C.M.Edin., appointed Visiting Physician to the Grafton Street and Parkhill Hospitals, Liverpool. A. Sykes, L.R.C.P.Lond., M.R.C.S.Eng., appointed Assistant Medical Officer to the City and Borough Asylum, Hellesdon, near Norwich. N. Williams, M.B.Cantab., M.R.C.S., re-appointed Medical Officer to the Harrogate Cottage Hospital.

### VACANCIES.

### Resident Medical Officers.

Central London Sick Asylum, Cleveland Street, Fitzroy Square, W., Assistant Medical Officer and Dispenser, doubly qualified, unmarried.
Huddersfield Infirmary, Senior Assistant House Surgeon.—Salary £50 per annum, with board, lodging, and washing.
Huddersfield Infirmary, Junior House Surgeon.—Salary £40 per annum, with board, lodging, and washing.
Ingham Infirmary and South Shields and Westoe Dispensary, House Surgeon, doubly qualified.—Salary £50 per annum, rising to £60.
Liverpool Eye and Ear Infirmary House Surgeon, doubly qualified.—Salary £80 per annum, with residence and maintenance.
Newport and County Infirmary, Newport, Mon., House Surgeon, doubly qualified.—Salary £100 per annum, with board and residence.
Norfolk County Asylum, Thorpe, Norwich, Medical Officer, single, and under 30 years of age, appointment for five years—Salary £160 per annum, with board, lodging, and washing.
Northumberland County Asylum, Morpeth, Clinical Clerk.—Board and residence provided.
Ramsgate and St. Lawrence Royal Dispensary and Seamen's Infirmary, Resident Medical Officer, doubly qualified, unmarried.—Salary £120 per annum, with furnished apartments, gas, firing, and attendance.
Tower Hamlets Dispensary, White Horse Street, Stepney, E., Resident Medical Officer.—Salary £120 per annum, with furnished rooms, coals, gas, and attendance.
West Bromwich District Hospital, House Surgeon, doubly qualified, unmarried.—Salary £80 per annum, with board, residence, and washing.

### Non-Resident Medical Officers.

Bradford Infirmary and Dispensary, Honorary Gynæcologist.
Cancer Hospital, Free, Brompton, S.W., two Surgeons; must be F.R.C.S.Eng., and reside within the four miles radius.
City of London Hospital for Diseases of the Chest, Victoria Park, E., Assistant Physician; must be Fellow or Member of the Royal College of Physicians, London.
County Down Infirmary, Surgeon.
County Kilkenny Infirmary, Second Surgeon—Salary £75 per annum.
Dental Hospital of London, Leicester Square, two Assistant Dental Surgeons; must be Licentiates in Dental Surgery.
Hospital for Consumption and Diseases of the Chest, Brompton, Assistant Physician.
Hospital for Diseases of the Throat, Golden Square.—Vacancy on the Honorary Medical Staff.

